# A Case Series and Literature Review of Vertebral Artery Stump Syndrome

**DOI:** 10.3389/fneur.2021.770845

**Published:** 2022-01-28

**Authors:** Wenbin Zhang, Shouchun Wang, Chao Li, Zhongxiu Wang, Feixue Yue, Jie Zhou, Kangjia Song, Chao Wang, Yujiao Wang, Mingchao Shi

**Affiliations:** ^1^Department of Neurology, The First Bethune Hospital of Jilin University, Jilin University, Changchun, China; ^2^Nursing Department, The First Bethune Hospital of Jilin University, Jilin University, Changchun, China

**Keywords:** vertebral artery stump syndrome, endovascular thrombectomy, tandem lesions, diagnosis, therapy

## Abstract

**Purpose:**

Stump syndrome refers to cerebral ischaemic symptoms caused by an embolus from a previously occluded ipsilateral artery that occludes a down-stream artery. It can be divided into two types: carotid stump syndrome and vertebral artery stump syndrome (VASS). At present, there is limited clinical experience with VASS. We aimed to propose a more precise diagnostic standard for VASS, and to share our experience with handling this condition.

**Materials and Methods:**

We retrospectively collected data of patients who were treated with endovascular thrombectomy in the stroke centre of the First Hospital of Jilin University from January 2016 to March 2020. After screening patients with posterior-circulation acute ischaemic stroke, we selected patients who had an acute occlusion of an intracranial artery in the context of a previously occluded ipsilateral vertebral artery origin, as confirmed by digital subtraction angiography.

**Results:**

Eleven patients met our inclusion criteria. Nine patients achieved recanalization of both extracranial and intracranial occluded arteries, one patient had modified thrombolysis in cerebral infarction grade 3, and eight patients had grade 2b. Residual stenosis of recanalized intracranial arteries was less than 30% in all cases, while three patients had embolism of distal arteries. No dissection or subarachnoid haemorrhage occurred. Two patients didn't reach vascular recanalization. Among the nine patients with recanalized artery, four had a 90-day modified Rankin Scale score ≤ 3 (favourable outcome), and four patients died; As for the two non-recanalized patients, one had a mRS score of 5 and one died.

**Conclusion:**

VASS is a clinical syndrome caused by embolic occlusion of a distal intracranial artery occluded ipsilateral extracranial vertebral artery. Antegrade blood flow from the collateral vessels, distal embolic occlusion and mild or no residual stenosis of the occluded intracranial artery after recanalization are notable features of this clinical event. Endovascular thrombectomy may be effective for treating VASS.

## Introduction

Carotid stump syndrome, a special and rare cerebral infarction syndrome, was first discussed in 1978 (1). Nguyen et al. (2) found similar symptoms in posterior-circulation acute ischaemic stroke and proposed a definition of vertebral artery stump syndrome in 2008. Currently, there is limited clinical data on VASS. In recent years, several studies (3–6) have reported on VASS (Table 1). However, most of the included cases did not have direct evidence of a cerebral infarction caused by emboli, and ischaemia may have been caused by hypoperfusion.

**Table 1:** Summary of previous VASS reports.

**Year**	**Authors**	**Clear definition or diagnostic criteria of VASS**	**Number of patients**	**Main treatment**	**Prevention**	**Outcome**
2007	Nguyen et al. (2)	No	2	Endovascular intervention, placing of coils	Warfarin or clopidogrel + aspirin	Good
2010	Kawano et al. (3)	No	3	Anticoagulation	Warfarin	Good
2012	Kawano et al. (4)	Yes	12	Anticoagulation as the basis	Clopidogrel, warfarin, aspirin, or aspirin + warfarin	Good
2018	Suzuki et al. (5)	Yes	1	Intravenous infusion	Clopidogrel	Good
2020	Maeoka et al. (6)	Yes	2	EVT	—	One good, one poor

*VASS, vertebral artery stump syndrome; EVT, endovascular thrombectomy*.

Therefore, we aimed to propose a more precise diagnostic standard for VASS and to share our experience managing this condition.

## Materials and Methods

### Patients

Among 211 patients with posterior-circulation acute ischaemic stroke treated with endovascular thrombectomy (EVT) at the stroke centre of our institution between January 2016 and March 2020, 11 patients met the following criteria: (i) acute ischaemic stroke in the posterior circulation; (ii) vertebral artery origin (VAO) occlusion identified either on magnetic resonance angiography, duplex ultrasonography, computed tomography (CT) angiography, or other conventional angiography, and at the same time presented with distal antegrade flow in the ipsilateral vertebral artery (VA); (iii) presence of an intracranial vascular occlusion distal to the extracranial occluded artery; and (iv) absence of other causes of ischaemic stroke, including intracranial vertebrobasilar lesions, hypercoagulable state, and arterial dissections. And the study design was approved by the appropriate ethics review board.

### Treatment Methods

**Figure 1:**
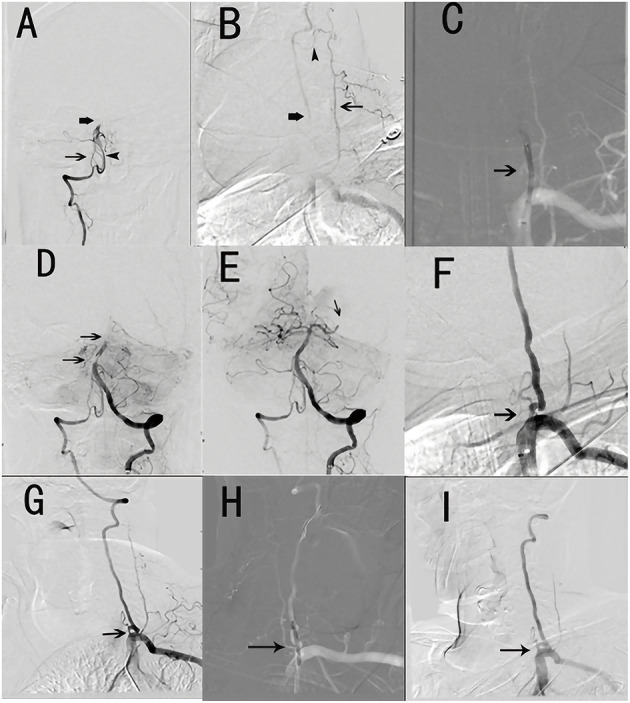
Typical surgical procedures (patient No. 4). **(A)** Hypoplasia of the right vertebral artery in the V4 segment (thin arrow) and clot within the proximal basilar artery (thick arrow) and posterior inferior cerebellar artery (arrowhead). **(B)** Left vertebral artery occlusion (thick arrow) and truncus thyrocervicalis (thin arrow) with collateral compensatory vessels (arrowhead). **(C)** Balloon dilation of left vertebral artery (small arrow). **(D)** Distal basilar artery clot (two arrows). **(E)** Recanalization of the basilar artery and embolized left posterior cerebral artery (small arrow). **(F)** The occlusion left vertebral artery turn to moderate stenosis (small arrow). **(G)** Severe ostial stenosis of the left vertebral artery 7 months after operation (small arrow). **(H)** Balloon dilation of the left vertebral artery (small arrow). **(I)** Severe ostial stenosis of the left vertebral artery turning to moderate stenosis (small arrow).

After the patient's relatives provided informed consent for operative treatment, EVT was performed under general anaesthesia. After implantation of a 6F or 8F arterial catheter through the femoral artery, cerebral DSA was performed to evaluate the vascular occlusion location and collateral status. If both VAOs were occluded, we chose the easier side as the approach; if the VA was occluded or underdeveloped unilaterally, we chose the dominant artery as the approach. After balloon dilation of the proximal VAO, we performed EVT for the distal occluded artery. EVT methods included mechanical thrombectomy (by stent retriever and aspiration catheter) and arterial thrombolysis. After distal vascular recanalization, stent implantation of the VAO would be considered necessary if the antegrade blood flow was intervened by either severe stenosis or unstable/ruptured plaque; otherwise, we ended the procedure. A typical case is illustrated in Figure 1. All patients received intravenous injection of 3,000 IU low molecular weight heparin calcium before surgery and 1,000 IU per h during the surgery. Patients were admitted to the neurological intensive care unit and completed a cerebral CT scan and dynamic electrocardiogram.

### Outcomes

Patients were administered with double-antiplatelet drug therapy orally for 6 months after discharge, followed by the administration of a single-antiplatelet drug. Assessment of the 90-day modified Rankin Scale (90d mRS) score was performed by experienced physicians in the Department of Neurology. Favourable outcome was defined as mRS score ≤ 3 with an mRS score of 6 indicating death.

## Results

### Demographic Characteristics

Eleven patients (10 men and 1 woman; mean age: 65.5 [range, 55–75] years) underwent EVT. The mean National Institutes of Health Stroke Scale (NIHSS) score was 23.5 (range, 5–35). Six patients (Nos. 1, 2, 5, 6, 8, 9) had a history of stroke, four patients (Nos. 1, 5, 7, 9) had coronary heart disease, and one patient (No. 5) had atrial fibrillation on dynamic electrocardiogram after EVT. All patients had at least one risk factor for atherosclerosis (e.g., hypertension, diabetes mellitus, history of stroke). All patients had a disturbance of consciousness of various degrees (Table 2).

**Table 2:** Baseline demographic characteristics.

**Patient** **number**	**Sex**	**History** **of stroke**	**HT**	**DM**	**Dyslipidaemia**	**AF**	**CHD**	**HHE**	**Smoking**	**Drinking**	**State of** **consciousness**	**OPT (h)**	**Pre-NIHSS**
1	M	+	+	–	+/↑	–	+	–	+	–	Somnolence	2	13
2	M	+	–	–	–	–	–	–	–	–	Subcoma	1.67	35
3	M	–	+	–	–	–	–	–	+	–	Subcoma	3.08	35
4	F	–	+	–	+/↑	–	–	–	–	–	Sopor	42.38	19
5	M	+	+	–	+/↓	–	+	–	+	+	Moderate coma	8.33	35
6	M	+	+	–	–	+	–	–	+	+	Somnolence	2.27	12
7	M	–	+	+	+/↓	–	+	–	+	–	Confusion	5.95	5
8	M	+	+	–	+/↑	–	–	+	–	+	Subcoma	161.83	35
9	M	+	+	–	+/↑	–	+	+	+	+	Somnolence	26.58	26
10	M	–	+	–	+/↓	–	–	–	–	+	Somnolence	2.33	9
11	M	–	+	+	+/↑	–	–	–	+	+	Sopor	11.5	35

*HT, hypertension; DM, diabetes mellitus; AF, atrial fibrillation; CHD, coronary heart disease; HHE, hyperhomocysteinaemia; OPT, onset-to-puncture time; NIHSS, National Institutes of Health Stroke Scale*.

### Surgical Procedures

Among the 11 patients treated with EVT, vascular recanalization of criminal vessel was achieved in nine patients (81.81%). Distal occlusive lesions did not achieve recanalization in two patients (Nos. 2, 11).

Regarding distal occlusions, embolized basilar arteries (BA) were seen in 10 patients and the occluded posterior cerebral artery was seen in one patient. Among the nine patients with successful recanalization, one patient (No. 1) was treated with arterial thrombolysis, while the other eight patients were treated with mechanical thrombectomy.

Regarding proximal occlusions, mere balloon angioplasty was performed at the occluded VAO in Two patients (Nos. 4, 7), while stents were placed in six patients (Nos. 1, 3, 5, 6, 8, 10). For patient No. 9, a stent was placed in the contralateral subclavian artery because of poor access to the ipsilateral VA, and the steal flow converted to antegrade blood flow. The embolus of the occluded BA lysed and the BA was successfully recanalized. Stents were placed at the occluded VAO in the remaining patients. Regarding stent types, balloon-mounted stents were used in all patients. Residual stenosis of the intracranial occluded lesions in the nine patients with vascular recanalization was <30%, as confirmed by DSA (Table 3).

**Table 3:** Endovascular treatment details and follow-up results.

**Patient number**	**Arterial lesion**	**Contralateral vertebral artery**	**Stump**	**Distal disposal**	**Proximal** **disposal**	**Residual** **stenosis <30%**	**Complication**	**Pre-mTICI**	**Post-mTICI**	**NIHSS at** **discharge**	**90d mRS**	**Recurrence**
1	LVAO RPCAO	Inferior, Occlusion after entering the skull	+	Arterial thrombolysis	BS	+	None	0	2b	5	3	^*^
2	Bil VAO BAO	Occlusion at the beginning	+ –	–	–	–	–	0	0	35	6	–
3	RVAO BAO	Occlusion after PICA	+	MT+aspiration	BS	+	–	0	2b	35	6	–
4	LVAO BAO	Inferior, V4 slim	–	MT+aspiration	BAG	+	Pneumonia	0	2b	8	1	Once
5	RVAO BAO	Occlusion after PICA	–	MT+aspiration	BS	+	Pneumonia	0	2b	25	6	–
6	LVAO BAO	Tortuous, V4 slim	+	MT+aspiration	BS	+	None	0	2b	0	0	Once
7	LVAO BAO	Occlusion after entering the skull	+	MT+aspiration	BAG	+	Pneumonia	0	2b	3	4	None
8	LVAO BAO	Occlusion after PICA	–	MT+aspiration	BS	+	Pneumonia	0	2b	35	6	–
9	RVAO BAO LSubASS	Inferior, Steal flow	+	MT+Aspiration	BS*	+	Hernia	0	3	35	6	–
10	LVAO BAO	V4 slim	+	MT+Aspiration	BS	+	None	0	2b	1	2	None
11	RVAO BAO	Inferior, From the aortic arch, slender and tortuous	–	Arterial thrombolysis	–	–	–	0	0	35	5	None

mTICI, modified thrombolysis in cerebral infarction; NIHSS, National Institutes of Health Stroke Scale; mRS, modified Rankin Scale; LVAO, left vertebral artery occlusion; RVAO, right vertebral artery occlusion; BAO, basilar artery occlusion; RPCAO, right posterior cerebral artery occlusion; LsubASS, left subclavian artery severe stenosis; Bil VAO, bilateral vertebral artery occlusion; MT, mechanical thrombectomy; BAG, balloon angioplasty; BS, balloon stent; BS*, the stent was placed at the subclavian artery; *lost to follow-up

* *lost to follow-up*.

### Post-operative Complications

Three patients developed distal embolization. No vascular dissection or subarachnoid haemorrhage occurred. One patient had a reperfusion injury, which developed into cerebral oedema, and the patient died. Four patients developed pulmonary infections, two of whom died of pulmonary infection combined with multiple organ failure (unrelated to the surgical procedures).

### Follow-Up

Among the nine patients with successful vascular recanalization, four had a 90d mRS score ≤ 3, one had an mRS score of 4, and four patients died. The rate of good outcome in patients with recanalization was 44.44%, and the mortality rate was 44.44%. Of the two non-recanalized patients, one had a 90d mRS score of 5 and one died. The rate of good outcome among all patients was 36.4%, and the mortality rate was 45.5%.

Among the six patients with an onset-to-puncture time (OPT) of <8 h, three patients had a 90d mRS score ≤ 3, one had a score of 4, and two patients died. Among the five patients with an OPT of >8 h, one had a 90d mRS score of 1, one had a score of 5, and three died.

Among six patients with a severe disturbance of consciousness and NIHSS score ≥26 before EVT, one patient had a 90d mRS score of 5 and five patients died. Among five patients with a mild disturbance of consciousness and NIHSS score ≤ 19 before EVT, four patients had a 90d mRS score ≤ 3 and one had a score of 4.

Patient No. 1 was lost to follow-up at 12 months after discharge but did not have a recurrence of stroke during the achieved follow-up. The remaining five patients have been followed up until the time of this submission. Three patients (Nos. 7, 10, 11) had no recurrence during the follow-up period, while two patients had stroke recurrence. Although patient No. 4 experienced dizziness and diplopia at the 6-month follow-up, CT did not show an apparent infarction, duplex ultrasound showed VA restenosis, and balloon dilation of the artery significantly alleviated the symptoms (Figure 1). Patient No. 6 had one episode of transient ischaemic attack (the lower limbs were disabled for a few minutes), and duplex ultrasound showed severe stenosis of the left VAO. However, the patient was not hospitalised because the symptoms were mild.

## Discussion

### Diagnostic Criteria of VASS

In 2008, Nguyen et al. (2) first proposed a definition of VASS, and in 2013, Kawano et al. (4) provided the following diagnostic criteria: (i) acute ischaemic stroke in the posterior circulation; (ii) VAO occlusion identified on magnetic resonance angiography, duplex ultrasound, CT angiography, and/or conventional angiography; (iii) presence of distal antegrade flow in the ipsilateral VA; and (iv) absence of other causes of ischaemic stroke, including intracranial vertebrobasilar arterial lesions or embolic diseases, which excludes arterial embolism. The 12 cases reported by Kawano et al. (4) did not show occlusion of posterior-circulation large arteries, such as the BA. Thus, stroke in these cases might have been caused by hypoperfusion. Other previous VASS reports (3–5) contained stroke cases that could also be caused by hypoperfusion. Because the existence of the embolus has not been confirmed, the existence of turbulence is only detected by ultrasound at most. Only two cases of VASS reported recently by Maeoka et al. (6) with distal BA occlusion were genuinely VASS. At the time of their report, it was difficult to identify whether intracranial artery occlusions were caused by embolism. However, interventional thrombectomy technology has become more developed, and currently, it is possible to determine whether intracranial arterial occlusion is caused by embolism. Thus, the diagnostic criteria of VASS require modification.

Based on the previous diagnostic criteria of VASS and our clinical experience, we propose diagnostic criteria for VASS more precise than those of Kawano et al. (4) as follows: (i) acute ischaemic stroke in the posterior circulation; (ii) VAO occlusion identified on magnetic resonance angiography, duplex ultrasound, CT angiography, and/or conventional angiography and the presence of distal antegrade flow in the ipsilateral VA; (iii) coexistence of an occluded intracranial artery distal to the occlusive extracranial artery; (iv) absence of other causes of ischaemic stroke (e.g., intracranial vertebrobasilar lesions, hypercoagulable state, or arterial dissections); and (v) residual stenosis of the intracranial artery <30% after EVT. Conformity with the first four diagnostic criteria is a strong indication of VASS, and cases meeting all five diagnostic criteria are considered confirmed VASS.

In patient No. 6, one of his VAs was occluded and the other VA was underdeveloped, even he had paroxysmal atrial fibrillation, it could not cause BA embolisation through the collateral circulation capillaries because of the different diameters. The contralateral vertebral artery was either occluded distal to the posterior inferior cerebellar artery, or the V4 segment was slender or occluded in the rest of the patients. Patient No. 9 had persistent steal flow. Other than embolism caused by vertebral artery occlusion, there was no valid explanation for intracranial artery embolism in these 11 cases.

Two of the five previous reports of VASS cases did not mention the diagnostic criteria. Among the 15 patients in the three articles mentioning the diagnostic criteria, 13 did not have clinical evidence of an intracranial vascular occlusion; only two cases were confirmed as intracranial artery occlusion by DSA, and only one case had no significant residual stenosis after recanalization (6). Therefore, only one case was considered confirmed VASS. In our report, nine patients with recanalization had no residual stenosis in the intracranial vessels after interventional therapy, and thrombosis due to arterial stenosis could be excluded. Emboli removed from the occluded intracranial artery also support the hypothesis that the emboli arose from the distal end of the extracranial occlusion, leading to cerebral embolism. Therefore, among the 11 patients treated with EVT, VASS could be diagnosed in the nine patients with recanalization. Two patients without successful recanalization met the former four diagnostic criteria which could be diagnosed as highly suspicious VASS.

### Treatment of VASS

Although the current preferred treatment method for acute ischaemic stroke within 4.5 h of onset is intravenous thrombolysis with recombinant tissue plasminogen activator (7), the recanalization rate of intravenous thrombolysis for arterial occlusion is still <50% (8). Additionally, the outcomes of intravenous thrombolysis for extracranial and intracranial arterial tandem lesions are thought to be more disappointing. VASS is a rare cerebral infarction syndrome, while drug therapy is preferred for patients with mild symptoms, EVT should be considered for patients whose symptoms are severe. Five patients in our study arrived at the hospital within 3.5 h after stroke onset. Because they had severe symptoms and disturbance of consciousness, only one patient underwent intravenous thrombolysis prior to EVT; all other patients underwent EVT directly.

The prognosis of patients with acute vertebrobasilar artery occlusion tended to be poor, with rates of mortality and disability reaching 68–77.7% (7, 9). Prompt recanalization of the occluded vessels and restoring cerebral perfusion is the basis of VASS treatment. EVT has been proven as the most effective treatment for acute ischaemic stroke caused by anterior-circulation arterial occlusion (10–15), and the time window has been extended to 16–24 h (16). Several studies have reported the effectiveness of EVT in the posterior circulation. The BEST study (17) analysed the effectiveness of different therapeutic methods and found that the favourable outcome rate was significantly higher in the EVT group (46.8%) than in the control group (24.1%) (*P* = 0.008). However, the high rate of medical therapy cases transferring to the EVT group may have affected the accuracy of the conclusion. Recently, the BASILAR study (9) revealed the effectiveness of EVT for posterior-circulation acute ischaemic stroke within 24 h of onset. Good outcome was significantly more frequent in the EVT group (32%) than in the control group (9.3%), and the mortality was lower in the EVT group (46.2%) than in the control group (71.4%).

Regarding the treatment of tandem lesions in VASS, a few case series have reported the safety and effectiveness of EVT for vertebrobasilar artery tandem lesions (18–21). One article reported two VASS cases with BA occlusion confirmed by DSA. One patient underwent successful recanalization and had a good outcome; however, the other patient did not achieve recanalization and had a poor outcome (6). The EVT recanalization rate of the 11 patients in the present study was 81.81%. Among the nine patients with recanalization, the rate of a good outcome was 44.44% and that of mortality was 44.44%. In the two non-recanalized patients, one patient had severe symptoms and one died. It seemed that EVT was one effective therapy for VASS and successful recanalization was an important factor for a good outcome. During the follow-up period, two patients with severe VAO restenosis had stroke recurrence with mild symptoms, while four patients did not experience recurrence. Recanalization of the proximal occluded artery and antiplatelet therapy can prevent recurrent emboli originating at the distal occlusion of the stump.

### Prognosis of VASS

Previously reported predictors of anterior-circulation EVT outcomes included the infarct volume, OPT, collateral circulation status, and occlusion aetiology (22–25). However, few studies have reported on the factors influencing outcomes for posterior-circulation lesions. Recently, Lee et al. (26) showed that when selecting patients with vertebrobasilar occlusion for endovascular treatment, an OPT of <8 h is predictive of a good outcome. In our study, among the six patients with an OPT of <8 h, three patients had a 90d mRS score ≤ 3, one patient had a score of 4, and two patients died. Among the five patients with an OPT of >8 h, one patient had a 90-d mRS score of 1, one had a score of 5, and three died. These results also support the concept that shortened OPT is favourable for a good outcome.

Six patients with severe disturbances of consciousness and NIHSS scores ≥26 had poor outcomes. Among the five patients with mild disturbances of consciousness and NIHSS scores ≤ 19 before EVT, four patients had good outcomes, and one had a poor outcome. We believe that a severe disturbance of consciousness and high NIHSS score before EVT are risk factors for a poor outcome. Among the five patients who died, recanalization was not achieved in one patient, and a reperfusion injury occurred in one patient, which developed into cerebral oedema, resulting in death. Additionally, one patient had a severe brainstem infarction before EVT, and two patients died of pulmonary infection after EVT. We speculate that the consciousness status and NIHSS score before EVT, as well as perioperative complications, directly affect the outcome.

### Limitations

Our study was based on data from a single centre, and the sample size was limited. Thus, multi-centre prospective trials are needed to obtain more conclusive results.

## Conclusions

Based on our findings on DSA and our experience of treating VASS by EVT, we conclude that VASS is a clinical syndrome caused by embolic occlusion of a distal intracranial artery occluded ipsilateral extracranial vertebral artery with notable features including antegrade blood flow from the collateral vessels, distal embolic occlusion and mild or no residual stenosis of the occluded intracranial artery after recanalization. Additionally, EVT may be an effective therapeutic method for VASS.

## Data Availability Statement

The raw data supporting the conclusions of this article will be made available by the authors, without undue reservation.

## Ethics Statement

The studies involving human participants were reviewed and approved by Ethics Committee of the First Hospital of Jilin University. The patients/participants provided their written informed consent to participate in this study.

## Author Contributions

MS contributed to the design of the article. CL is the key suggestions for the main issues of the article. WZ and SW made language polishing for the article. FY, JZ, KS, and YW conducted clinical data collection. All authors have read and approved the article.

## Conflict of Interest

The authors declare that the research was conducted in the absence of any commercial or financial relationships that could be construed as a potential conflict of interest.

## Publisher's Note

All claims expressed in this article are solely those of the authors and do not necessarily represent those of their affiliated organizations, or those of the publisher, the editors and the reviewers. Any product that may be evaluated in this article, or claim that may be made by its manufacturer, is not guaranteed or endorsed by the publisher.
